# Supplementary material for: Comparative analysis of molecular fingerprints in prediction of drug combination effects
Source: Brief Bioinform. 2021 Aug 17;22(6):bbab291. doi: 10.1093/bib/bbab291 (PMC8574997; doi:10.1093/bib/bbab291)
Supplement: supplementary_data_bbab291 [file supplementary_data_bbab291.pdf]

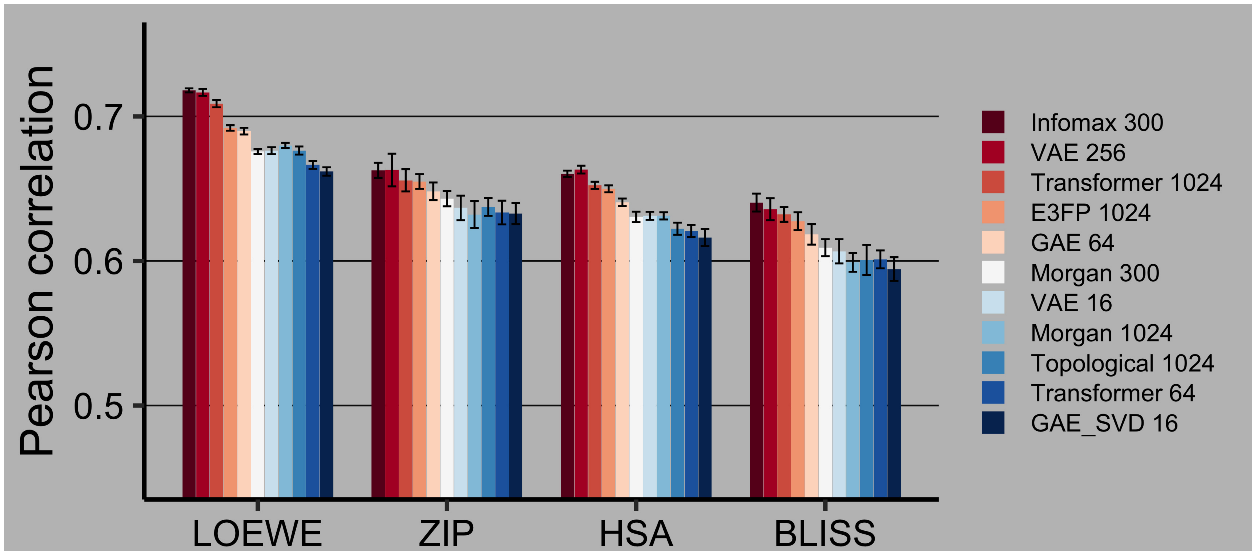

Figure S1: Drug combination synergy prediction on the CID-filtered dataset in 60 : 40 *train* : *test* split. 95% confidence intervals are calculated via Fisher z-transformation. Best models are highlighted with red. VS I task.

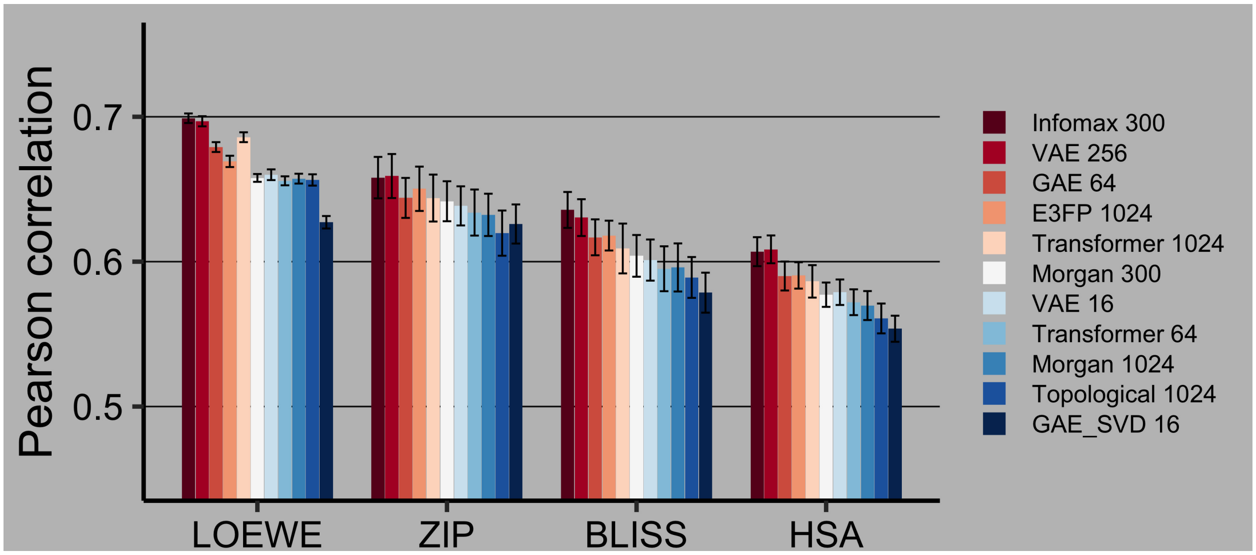

Figure S2: Drug combination synergy prediction on the SMILES-filtered dataset in 90 : 10 *train* : *test* split. 95% confidence intervals are calculated via Fisher z-transformation. Best models are highlighted with red. VS I task.

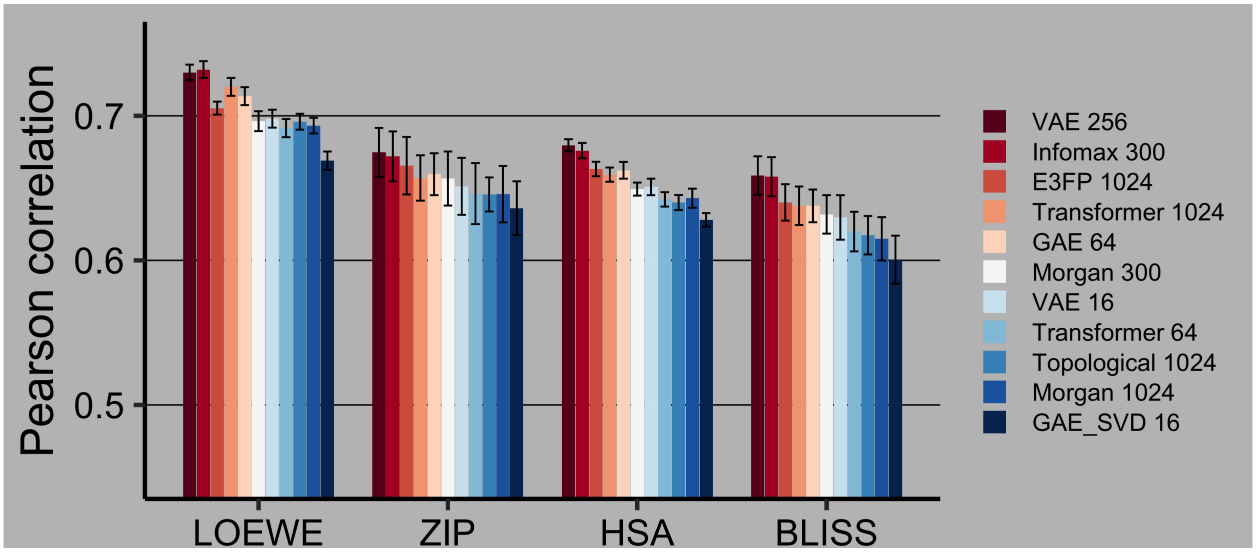

Figure S3: Drug combination synergy prediction on the CID-filtered dataset in 90 : 10 *train* : *test* split. 95% confidence intervals are calculated via Fisher z-transformation. Best models are highlighted with red. VS I task.

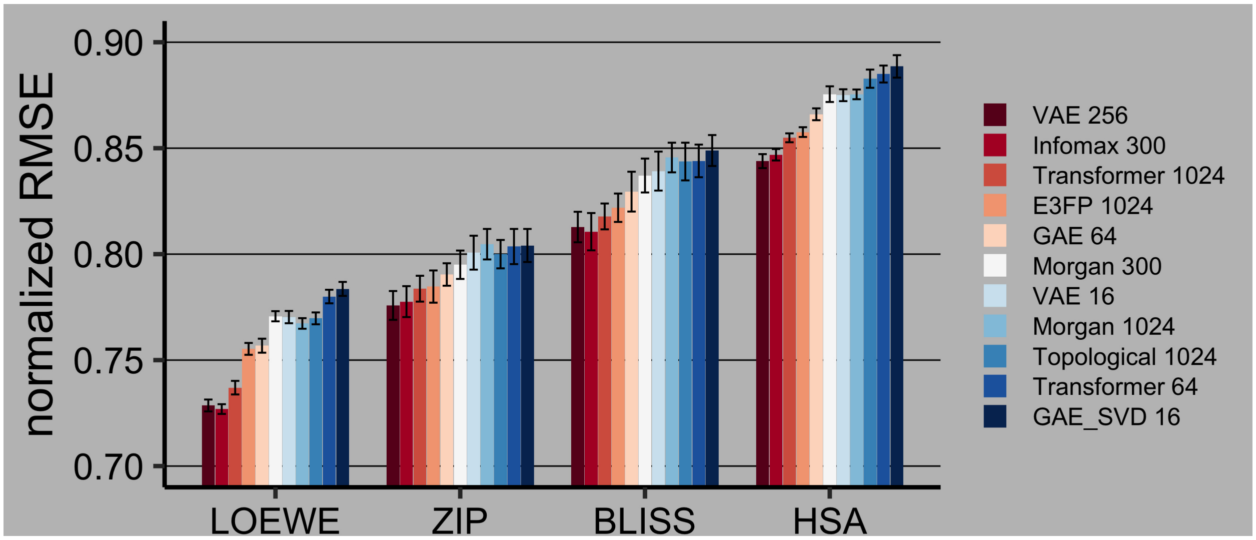

Figure S4: Drug combination synergy prediction on the CID-filtered dataset in 60 : 40 *train* : *test* split using RMSE, normalized by the target's standard deviation. 95% confidence intervals are calculated via empirical bootstrap. Best models are highlighted with red. VS I task.

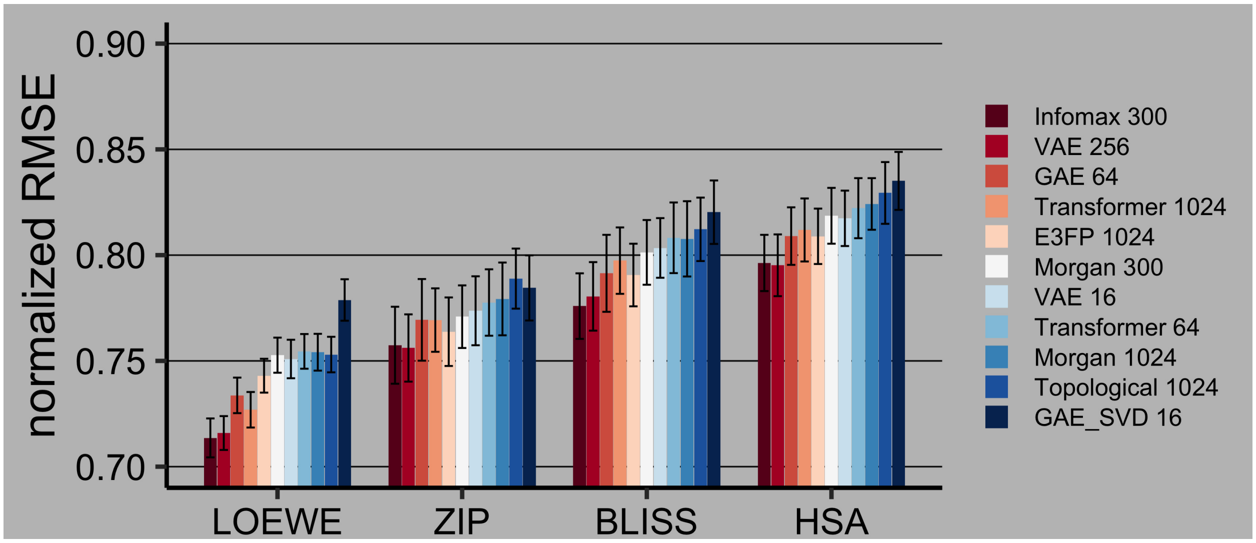

Figure S5: Drug combination synergy prediction on the SMILES-filtered dataset in 90 : 10 $train$: $test$ split using RMSE, normalized by the target's standard deviation. 95% confidence intervals are calculated via empirical bootstrap. Best models are highlighted with red. VS I task.

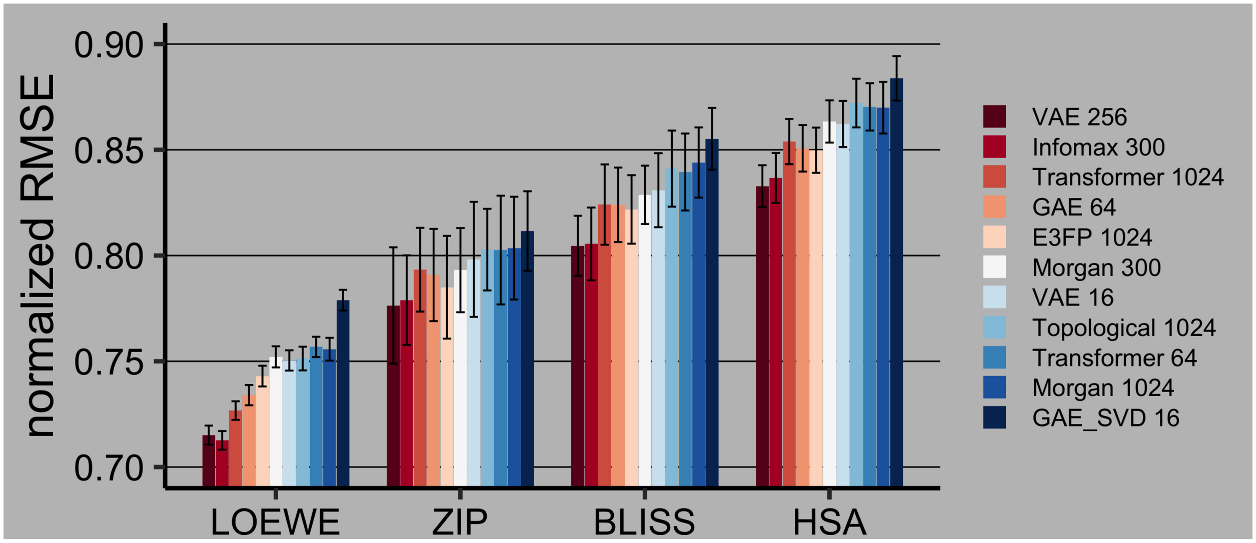

Figure S6: Drug combination synergy prediction on the CID-filtered dataset in 90 : 10 $train$: $test$ split using RMSE, normalized by the target's standard deviation. 95% confidence intervals are calculated via empirical bootstrap. Best models are highlighted with red. VS I task.

Table S1: Drug combination synergy prediction on the CID-filtered dataset in 60 : 40 *train* : *test* split. 95% confidence intervals are calculated via Fisher z-transformation. Best models are highlighted with bold. VS I task.

**fingerprint**	**Pearson's *r* and 95% Confidence Interval**				
	**CSS**	**Bliss**	**HSA**	**Loewe**	**ZIP**
E3FP 1024	0.8819 ±0.0019	0.6275 ±0.0062	0.6499 ±0.0024	0.6920 ±0.0019	0.6550 ±0.0051
GAE 16	0.8744 ±0.0018	0.5944 ±0.0082	0.6162 ±0.0059	0.6619 ±0.0029	0.6328 ±0.0073
GAE 64	0.8800 ±0.0022	0.6184 ±0.0071	0.6406 ±0.0020	0.6898 ±0.0023	0.6482 ±0.0061
Infomax 300	**0.8907 ±0.0016**	**0.6404 ±0.0062**	**0.6603 ±0.0022**	**0.7180 ±0.0014**	**0.6627 ±0.0052**
Morgan 300	0.8771 ±0.0017	0.6092 ±0.0059	0.6305 ±0.0036	0.6757 ±0.0017	0.6431 ±0.0053
Morgan 1024	0.8712 ±0.0022	0.5990 ±0.0065	0.6312 ±0.0025	0.6799 ±0.0018	0.6321 ±0.0093
Topological 1024	0.8718 ±0.0014	0.6007 ±0.0104	0.6223 ±0.0042	0.6764 ±0.0028	0.6374 ±0.0062
Transformer 64	0.8716 ±0.0014	0.6011 ±0.0062	0.6207 ±0.0042	0.6664 ±0.0027	0.6335 ±0.0082
Transformer 1024	**0.8854 ±0.0016**	**0.6322 ±0.0051**	**0.6523 ±0.0025**	**0.7088 ±0.0025**	**0.6558 ±0.0077**
VAE 16	0.8767 ±0.0016	0.6067 ±0.0084	0.6312 ±0.0028	0.6763 ±0.0024	0.6367 ±0.0085
VAE 256	**0.8908 ±0.0023**	**0.6358 ±0.0076**	**0.6632 ±0.0027**	**0.7166 ±0.0024**	**0.6629 ±0.0113**

Table S2: Drug combination synergy prediction on the SMILES-filtered dataset in 90 : 10 *train* : *test* split. 95% confidence intervals are calculated via Fisher z-transformation. Best models are highlighted with bold. VS I task.

**fingerprint**	**Pearson's *r* and 95% Confidence Interval**				
	**CSS**	**Bliss**	**HSA**	**Loewe**	**ZIP**
E3FP 1024	0.8732 ±0.0058	**0.6180 ±0.0103**	**0.5905 ±0.0091**	0.6692 ±0.0039	**0.6503 ±0.0153**
GAE 16	0.8641 ±0.0063	0.5786 ±0.0138	0.5537 ±0.0090	0.6272 ±0.0043	0.6260 ±0.0135
GAE 64	**0.8754 ±0.0064**	0.6168 ±0.0124	0.5901 ±0.0100	0.6791 ±0.0034	0.6440 ±0.0138
Infomax 300	**0.8826 ±0.0060**	**0.6357 ±0.0124**	**0.6069 ±0.0100**	**0.6990 ±0.0033**	**0.6580 ±0.0143**
Morgan 300	0.8702 ±0.0054	0.6040 ±0.0144	0.5772 ±0.0084	0.6578 ±0.0027	0.6417 ±0.0138
Morgan 1024	0.8640 ±0.0052	0.5960 ±0.0166	0.5697 ±0.0100	0.6573 ±0.0034	0.6323 ±0.0146
Topological 1024	0.8500 ±0.0044	0.5891 ±0.0141	0.5608 ±0.0103	0.6564 ±0.0039	0.6197 ±0.0156
Transformer 64	0.8682 ±0.0047	0.5951 ±0.0155	0.5720 ±0.0089	0.6558 ±0.0031	0.6339 ±0.0159
Transformer 1024	0.8742 ±0.0055	0.6091 ±0.0172	0.5864 ±0.0112	**0.6859 ±0.0034**	0.6439 ±0.0162
VAE 16	0.8712 ±0.0062	0.6011 ±0.0142	0.5789 ±0.0088	0.6600 ±0.0037	0.6385 ±0.0135
VAE 256	**0.8829 ±0.0063**	**0.6304 ±0.0127**	**0.6085 ±0.0096**	**0.6969 ±0.0035**	**0.6591 ±0.0152**

Table S3: Drug combination synergy prediction on the CID-filtered dataset in 90 : 10 *train* : *test* split. 95% confidence intervals are calculated via Fisher z-transformation. Best models are highlighted with bold. VS I task.

**fingerprint**	**Pearson's *r* and 95% Confidence Interval**				
	**CSS**	**Bliss**	**HSA**	**Loewe**	**ZIP**
E3FP 1024	0.8849 ±0.0049	**0.6401 ±0.0126**	**0.6632 ±0.0050**	0.7053 ±0.0045	**0.6655 ±0.0199**
GAE 16	0.8761 ±0.0041	0.6005 ±0.0166	0.6280 ±0.0047	0.6690 ±0.0063	0.6361 ±0.0186
GAE 64	**0.8869 ±0.0050**	0.6377 ±0.0113	0.6623 ±0.0058	0.7136 ±0.0062	0.6596 ±0.0145
Infomax 300	**0.8942 ±0.0056**	**0.6579 ±0.0135**	**0.6759 ±0.0053**	**0.7320 ±0.0058**	**0.6719 ±0.0173**
Morgan 300	0.8824 ±0.0045	0.6318 ±0.0133	0.6493 ±0.0045	0.6963 ±0.0069	0.6566 ±0.0187
Morgan 1024	0.8748 ±0.0053	0.6149 ±0.0150	0.6430 ±0.0066	0.6932 ±0.0054	0.6458 ±0.0195
TopoA 1024	0.8647 ±0.0049	0.6174 ±0.0133	0.6400 ±0.0052	0.6959 ±0.0055	0.6456 ±0.0118
TB 64	0.8798 ±0.0059	0.6199 ±0.0137	0.6422 ±0.0050	0.6915 ±0.0063	0.6462 ±0.0211
TB 1024	**0.8853 ±0.0047**	0.6378 ±0.0133	0.6593 ±0.0049	**0.7200 ±0.0062**	0.6570 ±0.0157
VAE 16	0.8838 ±0.0053	0.6297 ±0.0154	0.6508 ±0.0057	0.6980 ±0.0062	0.6512 ±0.0197
VAE 256	0.8949 ±0.0052	**0.6587 ±0.0133**	**0.6796 ±0.0042**	**0.7300 ±0.0054**	**0.6747 ±0.0169**

Table S4: Drug combination synergy prediction on the CID-filtered dataset in 60 : 40 *train* : *test* split using RMSE, normalized by the target's standard deviation. 95% confidence intervals are calculated via empirical bootstrap. Best models are highlighted with bold. VS I task.

**fingerprint**	**normalized Root Mean Squared Error and 95% Confidence Interval**				
	**CSS**	**Bliss**	**HSA**	**Loewe**	**ZIP**
E3FP 1024	0.4925 ±0.0014	0.8219 ±0.0067	0.8576 ±0.0023	0.7553 ±0.0028	0.7847 ±0.0076
GAE 16	0.4962 ±0.0014	0.8295 ±0.0094	0.8660 ±0.0028	0.7568 ±0.0033	0.7904 ±0.0053
GAE 64	0.5070 ±0.0015	0.8489 ±0.0073	0.8489 ±0.0073	0.7836 ±0.0033	0.8041 ±0.0078
Infomax 300	**0.4748 ±0.0011**	**0.8106 ±0.0088**	**0.8469 ±0.0027**	**0.7269 ±0.0023**	**0.7776 ±0.0073**
Morgan 300	0.5018 ±0.0013	0.8371 ±0.0080	0.8755 ±0.0037	0.7707 ±0.0024	0.7950 ±0.0067
Morgan 1024	0.5130 ±0.0016	0.8456 ±0.0070	0.8754 ±0.0023	0.7673 ±0.0025	0.8047 ±0.0072
Topological 1024	0.5118 ±0.0012	0.8437 ±0.0089	0.8828 ±0.0043	0.7697 ±0.0028	0.8000 ±0.0067
Transformer 64	0.5124 ±0.0014	0.8440 ±0.0077	0.8850 ±0.0040	0.7800 ±0.0032	0.8036 ±0.0083
Transformer 1024	**0.4855 ±0.0012**	**0.8178 ±0.0061**	**0.8549 ±0.0021**	**0.7370 ±0.0032**	**0.7837 ±0.0061**
VAE 16	0.5027 ±0.0013	0.8392 ±0.0092	0.8750 ±0.0028	0.7703 ±0.0029	0.8007 ±0.0080
VAE 256	**0.4744 ±0.0019**	**0.8128 ±0.0072**	**0.8439 ±0.0033**	**0.7286 ±0.0028**	**0.7758 ±0.0068**

Table S5: Drug combination synergy prediction on the SMILES-filtered dataset in 90 : 10 $train$: $test$ split using RMSE, normalized by the target's standard deviation. 95% confidence intervals are calculated via empirical bootstrap. Best models are highlighted with bold. VS I task.

fingerprint	normalized Root Mean Squared Error and 95% Confidence Interval				
	CSS	Bliss	HSA	Loewe	ZIP
E3FP 1024	0.4881 ±0.0033	**0.7906 ±0.0148**	**0.8089 ±0.0131**	0.7430 ±0.0080	**0.7638 ±0.0162**
GAE 16	0.5041 ±0.0041	0.8203 ±0.0150	0.8351 ±0.0137	0.7788 ±0.0098	0.7845 ±0.0154
GAE 64	**0.4842 ±0.0037**	0.7914 ±0.0182	0.8090 ±0.0136	0.7337 ±0.0084	0.7694 ±0.0193
Infomax 300	**0.4708 ±0.0038**	**0.7759 ±0.0155**	**0.7963 ±0.0133**	**0.7136 ±0.0092**	**0.7574 ±0.0182**
Morgan 300	0.4935 ±0.0033	0.8013 ±0.0153	0.8186 ±0.0132	0.7527 ±0.0083	0.7709 ±0.0148
Morgan 1024	0.5045 ±0.0039	0.8077 ±0.0178	0.8242 ±0.0122	0.7541 ±0.0087	0.7793 ±0.0172
Topological 1024	0.5276 ±0.0033	0.8122 ±0.0150	0.8294 ±0.0146	0.7530 ±0.0084	0.7889 ±0.0142
Transformer 64	0.4972 ±0.0034	0.8082 ±0.0167	0.8222 ±0.0142	0.7545 ±0.0082	0.7776 ±0.0157
Transformer 1024	0.4864 ±0.0033	0.7974 ±0.0157	0.8119 ± 0.0149	**0.7269 ±0.0084**	0.7693 ±0.0150
VAE 16	0.4919 ±0.0036	0.8034 ±0.0141	0.8174 ±0.0131	0.7509 ±0.0091	0.7737 ±0.0163
VAE 256	**0.4701 ±0.0037**	**0.7805 ±0.0162**	**0.7952 ±0.0146**	**0.7159 ±0.0080**	**0.7561 ±0.0159**

Table S6: Drug combination synergy prediction on the CID-filtered dataset in 90 : 10 $train$: $test$ split using RMSE, normalized by the target's standard deviation. 95% confidence intervals are calculated via empirical bootstrap. Best models are highlighted with bold. VS I task.

fingerprint	normalized Root Mean Squared Error and 95% Confidence Interval				
	CSS	Bliss	HSA	Loewe	ZIP
E3FP 1024	0.4871 ±0.0033	**0.8218 ±0.0162**	**0.8498 ±0.0107**	0.7430 ±0.0049	**0.7850 ±0.0243**
GAE 16	0.5043 ±0.0027	0.8552 ±0.0146	0.8838 ±0.0105	0.7789 ±0.0049	0.8116 ±0.0188
GAE 64	**0.4832 ±0.0030**	0.8240 ±0.0176	0.8507 ±0.0110	0.7340 ±0.0048	0.7908 ±0.0218
Infomax 300	**0.4683 ±0.0032**	**0.8055 ±0.0172**	**0.8367 ±0.0118**	**0.7126 ±0.0044**	**0.7789 ±0.0212**
Morgan 300	0.4920 ±0.0028	0.8287 ±0.0138	0.8634 ±0.0100	0.7521 ±0.0050	0.7931 ±0.0199
Morgan 1024	0.5068 ±0.0038	0.8440 ±0.0166	0.8699 ±0.0122	0.7557 ±0.0054	0.8035 ±0.0243
Topological 1024	0.5254 ±0.0033	0.8411 ±0.0180	0.8721 ±0.0115	0.7513 ±0.0056	0.8028 ±0.0193
Transformer 64	0.4972 ±0.0036	0.8395 ±0.0182	0.8703 ±0.0112	0.7568 ±0.0048	0.8026 ±0.0257
Transformer 1024	0.4863 ±0.0029	0.8241 ±0.0190	0.8539 ±0.0107	**0.7267 ±0.0044**	0.7933 ±0.0198
VAE 16	0.4895 ±0.0030	0.8309 ±0.0175	0.8622 ±0.0109	0.7504 ±0.0048	0.7982 ±0.0272
VAE 256	**0.4665 ±0.0032**	**0.8046 ±0.0142**	**0.8328 ±0.0099**	**0.7151 ±0.0045**	**0.7764 ±0.0275**
