# Supplementary material for: Comparative analysis of molecular fingerprints in prediction of drug combination effects
Source: Brief Bioinform. 2021 Aug 17;22(6):bbab291. doi: 10.1093/bib/bbab291 (PMC8574997; doi:10.1093/bib/bbab291)
Supplement: Supplementary_Figures_and_Tables_forfinal_review_bbab291 [file supplementary_figures_and_tables_forfinal_review_bbab291.docx]

Supplementary Figures and Tables

[Figure S1 location]

Figure S1. Drug combination synergy prediction on the CID-filtered dataset in 60:40 train:test split. 95% confidence intervals are calculated via Fisher z-transformation. Best models are highlighted with red.  VS I task.

[Figure S2 location]

Figure S2. Drug combination synergy prediction on the SMILES-filtered dataset in 90:10 train:test split. 95% confidence intervals are calculated via Fisher z-transformation. Best models are highlighted with red. VS I task.

[Figure S3 location]

Figure S3. Drug combination synergy prediction on the CID-filtered dataset in 90:10 train:test split. 95% confidence intervals are calculated via Fisher z-transformation. Best models are highlighted with red. VS I task.

[Figure S4 location]

Figure S4. Drug combination synergy prediction on the CID-filtered dataset in 60:40 train:test split using RMSE, normalized by the target’s standard deviation. 95% confidence intervals are calculated via empirical bootstrap. Best models are highlighted with red. VS I task.

[Figure S5 location]
Figure S5. Drug combination synergy prediction on the SMILES-filtered dataset in 90:10 train:test split using RMSE, normalized by the target’s standard deviation. 95% confidence intervals are calculated via empirical bootstrap. Best models are highlighted with red. VS I task.
[Figure S6 location]
Figure S6. Drug combination synergy prediction on the CID-filtered dataset in 90:10 train:test split using RMSE, normalized by the target’s standard deviation. 95% confidence intervals are calculated via empirical bootstrap. Best models are highlighted with red. VS I task.

Table S1. Drug combination synergy prediction on the CID-filtered dataset in 60:40 train:test split. 95% confidence intervals are calculated via Fisher z-transformation. Best models are in bold. VS I task.

[Table S1 location]

Table S2. Drug combination synergy prediction on the SMILES-filtered dataset in 90:10 train:test split. 95% confidence intervals are calculated via Fisher z-transformation. Best models are in bold. VS I task.

[Table S2 location]

Table S3. Drug combination synergy prediction on the CID-filtered dataset in 90:10 train:test split. 95% confidence intervals are calculated via Fisher z-transformation. Best models are in bold. VS I task.

[Table S3 location]

Table S4. Drug combination synergy prediction on the CID-filtered dataset in 60:40 train:test split using RMSE, normalized by the target’s standard deviation. 95% confidence intervals are calculated via empirical bootstrap. Best models are in bold. VS I task.

[Table S4 location]

Table S5. Drug combination synergy prediction on the SMILES-filtered dataset in 90:10 train:test split using RMSE, normalized by the target’s standard deviation. 95% confidence intervals are calculated via empirical bootstrap. Best models are in bold. VS I task.

[Table S5 location]

Table S6. Drug combination synergy prediction on the CID-filtered dataset in 90:10 train:test split using RMSE, normalized by the target’s standard deviation. 95% confidence intervals are calculated via empirical bootstrap. Best models are in bold. VS I task.

[Table S6 location]
